# Supplementary material for: Effect Analysis of Hydrogen Peroxide Using Hyperspectral Reflectance in Sorghum [Sorghum bicolor (L.) Moench] under Drought Stress
Source: Plants (Basel). 2023 Aug 16;12(16):2958. doi: 10.3390/plants12162958 (PMC10459410; doi:10.3390/plants12162958)
Supplement: Supplementary file 1 [file plants-12-02958-s001.zip › plants-2540001-supplementary.pdf]

Table S1. Description of chlorophyll fluorescence parameters.

Fluorescence parameters	Description
**Extracted parameter**	
O	At full photochemical quenching under dark-adapted conditions
T_{100}	Fluorescence intensity at 100 µs
K	Fluorescence intensity at 300 µs
J	Fluorescence intensity at 2 ms
I	Fluorescence intensity at 30 ms
P	=Fm
S	In the 30-100 s time domain and usually ascribed to
M	Non-photochemical quenching (NPQ)
Fo	Fluorescence intensity at 50 µs or 20 µs
Fm	Maximal fluorescence intensity
Fv	Fv = Fm - Fo
tFm	The time to reach Fm
**Selected OJIP parameters**	
Vj	Relative variable fluorescence at 2ms
Fv/Fo	The ratio of the variable fluorescence to Fo
Mo	The initial slope of the induction curve.
**Quantum efficiencies or flux ratios**	
ETo/ABS	The quantum yield of electron transport, the probability that an absorbed photon leads to an electron transport further than Q_A
Fv/Fm	The quantum yield of primary photochemistry.
ETo/Tro	The probability that trapped exciton moves an electron into the electron transport chain further than Q_A^-
**Area above the induction curve**	
Area	The total complementary area from time 0 to tFm
ECo/RC	Total electron carriers per RC
**Performance index**	
PI	Performance index. The energy conservation efficiency from absorbed photons to the reduction of intersystem electron carriers.
**Specific fluxes per active PS II reaction center**	
ABS/RC	Absorption flux, effective antenna size of an active reaction center.
TRo/RC	The maximal specific trapping flux
ETo/RC	Electron transport flux further than Q_A
DIo/RC	Dissipation flux.
**Phenomenological fluxes or activities per excited cross-section**	
ETo/CS	The relative number of actives PS II reaction centers per excite cross-section.
DIo/CS	Energy dissipation flux per excite cross-section at Fo
RC/CSm	Amount of active PS II RCs per CS at Fm
RC/CSo	Amount of active PS II RCs per CS at Fo
